# CMSA: a heterogeneous CPU/GPU computing system for multiple similar RNA/DNA sequence alignment

**DOI:** 10.1186/s12859-017-1725-6

**Published:** 2017-06-24

**Authors:** Xi Chen, Chen Wang, Shanjiang Tang, Ce Yu, Quan Zou

**Affiliations:** School of Computer Science and Technology, Tianjin University, Yaguan Road, Tianjin, China

**Keywords:** Heterogeneous, GPU, Multiple sequence alignment (MSA), Center star alignment

## Abstract

**Background:**

The multiple sequence alignment (MSA) is a classic and powerful technique for sequence analysis in bioinformatics. With the rapid growth of biological datasets, MSA parallelization becomes necessary to keep its running time in an acceptable level. Although there are a lot of work on MSA problems, their approaches are either insufficient or contain some implicit assumptions that limit the generality of usage. First, the information of users’ sequences, including the sizes of datasets and the lengths of sequences, can be of arbitrary values and are generally unknown before submitted, which are unfortunately ignored by previous work. Second, the center star strategy is suited for aligning similar sequences. But its first stage, center sequence selection, is highly time-consuming and requires further optimization. Moreover, given the heterogeneous CPU/GPU platform, prior studies consider the MSA parallelization on GPU devices only, making the CPUs idle during the computation. Co-run computation, however, can maximize the utilization of the computing resources by enabling the workload computation on both CPU and GPU simultaneously.

**Results:**

This paper presents CMSA, a robust and efficient MSA system for large-scale datasets on the heterogeneous CPU/GPU platform. It performs and optimizes multiple sequence alignment automatically for users’ submitted sequences without any assumptions. CMSA adopts the co-run computation model so that both CPU and GPU devices are fully utilized. Moreover, CMSA proposes an improved center star strategy that reduces the time complexity of its center sequence selection process from *O*(*mn* ^2^) to *O*(*mn*). The experimental results show that CMSA achieves an up to 11× speedup and outperforms the state-of-the-art software.

**Conclusion:**

CMSA focuses on the multiple similar RNA/DNA sequence alignment and proposes a novel bitmap based algorithm to improve the center star strategy. We can conclude that harvesting the high performance of modern GPU is a promising approach to accelerate multiple sequence alignment. Besides, adopting the co-run computation model can maximize the entire system utilization significantly. The source code is available at https://github.com/wangvsa/CMSA.

## Background

Multiple sequence alignment (MSA) refers to the problem of aligning three or more sequences with or without inserting gaps between the symbols [1]. It is a fundamental tool for similar sequences analysis in computational biology and molecular function prediction. In computational molecular biology, similar DNA sequences are aligned to find out the single nucleotide polymorphism and the copy-number variant, which is the key content in genetics [2]. In molecular function prediction, large-scale similar DNA sequence alignment is required when addressing the evolutionary analysis of bacterial and viral genomes [3]. Therefore, MSA software need to be efficient and scalable to handle large-scale datasets, which may contain hundreds of thousands of similar sequences.

MSA is a problem with an exponential time complexity, it has been proven to be NP-complete [4]. Many heuristic algorithms are developed and implemented by previous studies, including Kalign [5], MAFFT [6] and Clustal [7]. However, our experiments show that none of these heuristic-based softwares can address the alignment of large-scale RNA/DNA datasets with more than 100,000 sequences. Besides, all of these softwares are optimized either for short sequences or long sequences and none of them are designed for any arbitrary lengths of sequences.

On the other hand, heuristic methods model the MSA problem as multiple pairwise alignment problems, and there are two kinds of classic algorithms, i.e., tree-based algorithm and center star algorithm. In the tree-based algorithm, an evolutionary tree may be assumed, with the input sequences assigned to the leaves. Additional reconstructed sequences, corresponding to the internal nodes of the tree, are added to the multiple alignment. A star-alignment will denote the special case in which the tree has only one internal node. This is a reasonable model for the evolutionary history of some input sequences where all the observed sequences are assumed to have arisen by separate lineages from a single ancestral sequence [8]. For this scenario, the center star algorithm reduce the times of pairwise alignment, and both methods could achieve a similar accuracy. So in this paper, we focus on paralleling and optimizing the center star algorithm. A K-band strategy [2, 9] is proposed to reduce the time and space cost of dynamic programming process and then developed a MSA software named HAlign, which is based on the center star algorithm and K-band strategy. But its time complexity of finding the center sequence is still too high to make it practical for large-scale datasets. Therefore, we believe that it is necessary to further optimize the center star algorithm for large-scale MSA problems.

Recently, Graphic Processing Unit (GPU) with the Compute Unified Device Architecture (CUDA) programming model is widely used as additional accelerators for time-consuming computations. And heterogeneous CPU and GPU platform is a desirable way to overlap the computation of the CPU and GPU to fully exploit the compute capability and shorten the runtime [10]. However, in the multiple similar sequence alignment area, few parallel implementations exist that can address large-scale datasets and produce good speedups.

In this paper, we present CMSA, a robust and efficient MSA system for large-scale datasets on the heterogeneous CPU/GPU platform. CMSA is based on the center star strategy and mainly focuses on the alignment of similar RNA/DNA sequences. It can perform and optimize multiple sequence alignment automatically for users’ submitted sequences of any arbitrary length and volume. Second, it adopts the co-run computation model that leverages both CPU and GPU for sequence alignment. So it could maximize the entire system utilization. A pre-computation mechanism is developed in CMSA to estimate the computing capacity of CPU and GPU in advance. CMSA then distributes the workloads for CPU and GPU based on this estimation to achieve a better load balance. Furthermore, we propose a novel bitmap based algorithm to find the center sequence, which is the most crucial procedure in the center star strategy. The new algorithm reduces the time complexity from *O*(*mn* ^2^) to *O*(*mn*) without sacrificing the accuracy. The experiments demonstrate the efficiency and scalability of CMSA. Specifically, it shows that CMSA has a linear scalability as the number of sequences increases and achieves a speedup up to 11×. We also compare CMSA with the state-of-the-art MSA tools including MAFFT, Kalign, and HAlign. The results show that CMSA is much faster than these tools and is able to process large-scale datasets in an acceptable time, whereas previous tools cannot.

### Multiple similar sequence alignment

Similar sequences probably have the same ancestor, share the same structure, and have a similar biological function. The biological information associated with similar sequences can provide the necessary foundation for determining the structure and function of the newly discovered ones. For example, in computational molecular biology, the alignment of similar DNA sequences can be used to find single nucleotide polymorphism.

There are several MSA methods that utilize the feature of the similarity between sequences. Progressive MSA methods align the most similar sequences firstly, add then add the less related sequences to the alignment in succession. The basic idea is that long common substrings can be rapidly extracted from the pairwise sequences when the input sequences are highly similar. Thus, we only need to align the remaining short regions. However, few MSA tools are implemented for massive similar sequences alignment. Therefore, we need some methods to solve the MSA problem on similar large-scale datasets.

### Center star strategy

The main approach underlying the center star strategy is to transform the MSA problem into pairwise alignment problems.

For a dataset of *n* sequences with the average length of *m*, the *i* *th* sequence is define as *s*_*i*_, where 1≤*i*≤*n*. *S*_*ij*_ is the similarity score of sequences *s*_*i*_ and *s*_*j*_. *S*_*i*_ is the total similarity score of sequence *s*_*i*_. Then the *S*_*i*_ is can be computed with the following equation:

$$S_{i} = \sum_{j=1}^{n} S_{{ij}}, j \neq i $$

Center star strategy contains three steps:

- **Step 1. Center sequence selection**. Compute the total similarity score *S*_*i*_ for each sequence and choose the one with a maximum value as the center sequence.
- **Step 2. Pairwise alignment.** The center sequence then is pairwise aligned with each of the other sequences.
- **Step 3. Subtotaling the inserted spaces.** All of the inserted spaces are summed to obtain the final MSA result.

Now we analyze the time complexity of the center star strategy. The result is shown in Table 1. Suppose we use a dynamic programming method such as Needleman-Wunsch [11] to align sequences, which demands *O*(*m* ^2^) for both time and space. And in the first step, a naive way to find the center sequence is to align each pair of sequences, which costs a total time of *O*(*m* ^2^ *n* ^2^). Then in the second step, aligning the center sequence with other *n*−1 sequences demands a total time of (*mn* ^2^). The position information of all inserted gaps can be stored in *O*(*mn*) spaces. In the last step, those gaps are summed to generate the final result.

**Table 1:** The time complexity of the center star strategy

Step	Naive center star	HAlign	CMSA
1	*O*(*m* ^2^ *n* ^2^)	*O*(*mn* ^2^)	*O*(*mn*)
2	*O*(*m* ^2^ *n*)	*O*(*m* ^2^ *n*)	*O*(*m* ^2^ *n*)
3	*O*(*mn*)	*O*(*mn*)	*O*(*mn*)

The second column in Table 1 shows these three steps’ time complexity of a naive center star strategy. A conclusion can be drawn from the table that most of the time would be used to find the center sequence. To reduce this cost, HAlign [9] uses *trie trees* to accelerate the process. The time complexity for building a trie tree for one sequence is *O*(*m*). Searching *n* sequences in a trie tree incurs a time cost of *O*(*mn*). These two steps are performed *n* times to find the center sequence, which requires a total time of *O*(*mn* ^2^). But for large-scale datasets where *n*≫*m*, it’s still not efficient enough. Therefore, in this paper, we propose a novel bitmap-based algorithm that could reduce the time complexity of the first stage to *O*(*mn*) and also achieves a better accuracy compared to HAlign. Our approach will be discussed in detail in “An improved center star algorithm” section.

### Heterogeneous CPU/GPU architecture

There are several different parallel programming approaches on multi-core systems:

(i) Low-level multi-tasking or multi-threading such as POSIX Thread (pThread) library [12].
(ii) High-level libraries, such as Intel Threading Building Blocks [13], which provides certain abstractions and features attempting to simplify concurrent software development.
(iii) Programming languages or language extensions developed specifically for concurrency, such as OpenMP [14].

Moreover, GPU now is widely used to accelerate time-consuming tasks. GPU contains a scalable array of multi-threaded processing units known as streaming multi-processors (SM). Although GPU is originally designed to render graphics, general-purpose GPU (GPGPU) breaks this limit, and CUDA [15] is proposed as a general-purpose programming model for writing highly parallel programs. This model has proven quite successful at programming a broad range of scientific applications [16].

**Fig. 1:**
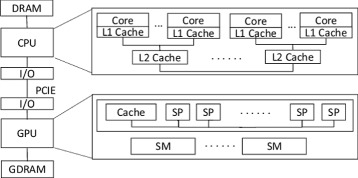
The heterogeneous CPU/GPU architecture. To achieving the best performance, the co-run model of CPU and GPU is adopted
(ii) CPU still plays the role of a master and at the same time, it shares a portion of GPU’s computations.

A heterogeneous CPU/GPU platform is proposed to achieve the best performance. Figure 1 depicts this architecture. CPU and GPU are connected by PCIE and both of them have their own memory space. There are two main methods for heterogeneous CPU/GPU programming.

(i) Consider CPU as a master and GPU as a worker. CPU handles the work assignment, data distribution, etc. GPU is responsible for the whole computation.

The former method has a clear work division between CPU and GPU but wastes the computing resource of CPU regrettably. The latter method has a better performance, but it also brings in some tricky issues such as the load balance and extra communications between CPU and GPU.

## Challenges and approaches

There are several key issues that we need to address for MSA in practice. In the following, we highlight these challenges and then give our corresponding solutions. The detailed implementation will be described in “Implementation” section.

**The MSA problem on similar RNA/DNA sequence.** Most MSA methods and tools ignore the similarity of RNA/DNA sequences, which is an important characteristic in RNA/DNA sequence alignment. Center star strategy is more suited for similar sequence alignment, but its center sequence selection process is very slow especially for large-scale datasets.

*An improved center star algorithm.* We have analyzed that the first stage, center sequence selection, is the most time-consuming part of a straightforward implementation of the center star strategy. Therefore, we designed a bitmap liked algorithm to find the center sequence. The time complexity is reduced from *O*(*mn* ^2^) to *O*(*mn*), as discussed in “Center star strategy” section.

**Low utilization problem on the heterogeneous CPU/GPU platform.** To further improve the performance of CMSA, parallelization is a sensible way. However, most GPU based MSA systems perform all computations on GPU only. The CPU source is idle. And these GPU based systems cannot run on different platforms which only contain CPU device. Therefore, it’s necessary to exploit the computing power of CPU and GPU at the same time.

*Co-run computation model.* To fully utilize all available computing capacities in the heterogeneous CPU/GPU platform, it is crucial to enable CPU and GPU work concurrently for workload computations (i.e., co-run computation), which means that CPU also performs a portion of computation instead of waiting for GPU to do all the work. The software designed for heterogeneous CPU/GPU platform can adapt to different computation environment. And when the GPU is not available, the CPU can handle the overall computation. Thus, CMSA can run on different platforms with or without GPUs. We designed a pre-computation mechanism to decide how to distribute workloads between CPU and GPU.

**Different lengths of sequences.** Previous MSA software mainly focus on either short or long sequences, but no work consider both of them.

*Automatical configuration.* CMSA could automatically configure the parameters like thread number and block number according to the lengths of sequences. When the space requirement exceeds GPU’s global memory limit, the related computation will be executed on CPU only.

## Implementation

In this section, the execution overflow of CMSA is first explained. Then our improved center star algorithm is discussed. At last, the implementation details of CMSA on the heterogeneous CPU/GPU platform is described.

### Execution overflow

**Fig. 2:**
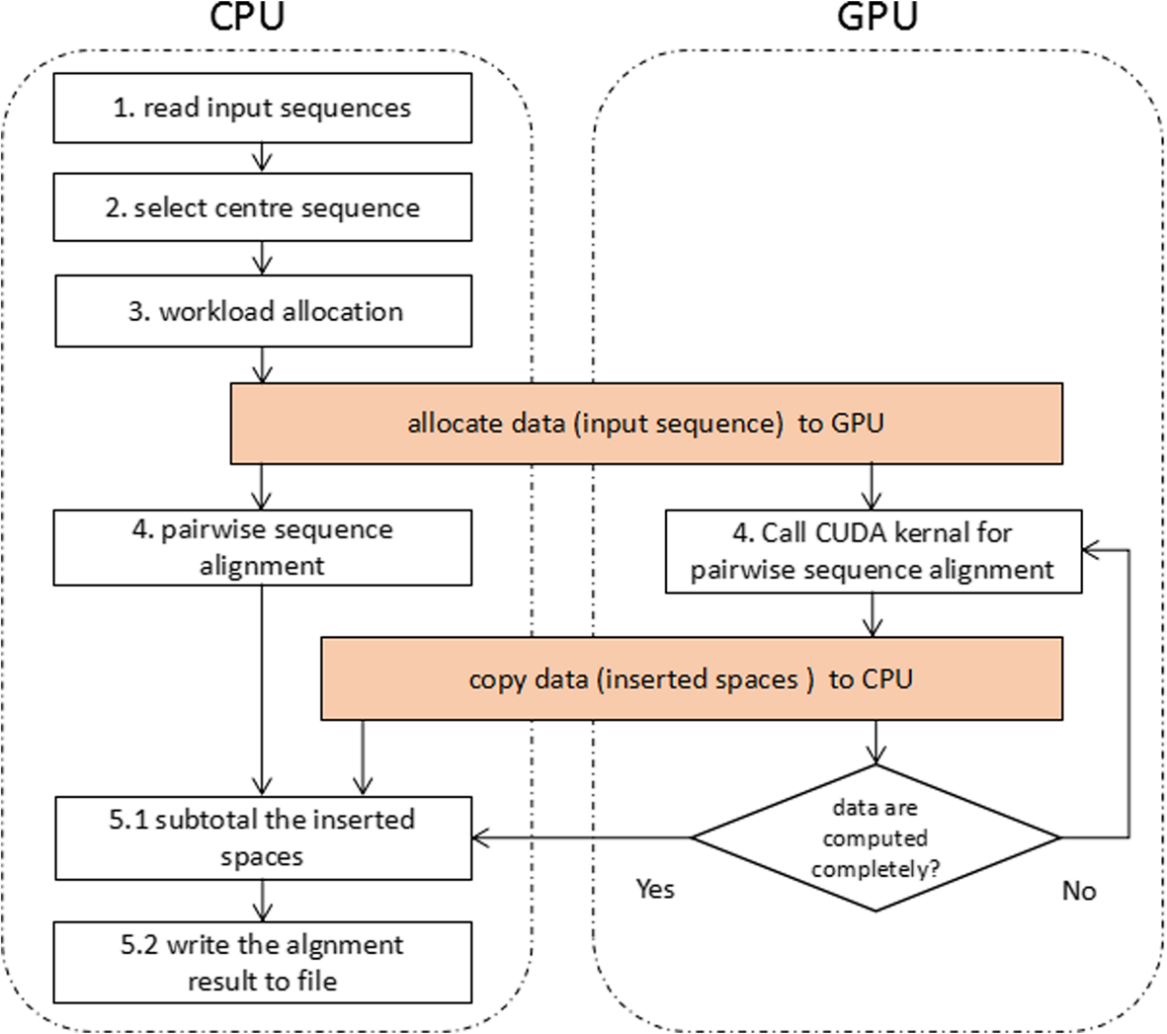
The overall flow of CMSA. Multiple sequence alignment is handled on the heterogeneous CPU/GPU platform
- **Step 2. Select center sequence.** We design a bitmap based algorithm to find the center sequence. This process has a time complexity of *O*(*mn*) and could be finished within few seconds even with massive sequences, so it is performed on CPU only without any parallelization. The algorithm will be discussed in “An improved center star algorithm” section.
- **Step 3. Workload allocation.** CMSA performs a pre-computation process to decide how to distribute workload for CPU and GPU. In this process, a small number of sequences are aligned in advance to evaluate the computing capacity of CPU and GPU. The detailed information of workload allocation will be described in “Workload distribution” section.
- **Step 4. Pairwise sequence alignment.** CPU and GPU independently execute pairwise alignment of assigned sequences. For better performances, tasks on CPU are executed in parallel by using OpenMP library. On the GPU end, the parameters like the number of threads in a block and the number of blocks in a grid can be automatically configured based on the different lengths of sequences. “Parallel optimization of pairwise alignment” section will describe the implementation of both ends.
- **Step 5. Output.** When both CPU and GPU finish their job, CMSA gathers the result from GPU and CPU, then merges the inserted gaps to generate the final result.

CMSA is a heterogeneous CPU/GPU system, using CUDA and OpenMP for parallelization. To reduce the total execution time, CPU also carries out part of the alignment task instead of waiting for GPU to deal with the whole work. The execution overview of CMSA is shown in Fig. 2. It contains following steps:

- **Step 1. Read input sequences.** CMSA reads all sequences into the host (CPU) memory. After the pre-computation process, a copy of sequences that would be handled by GPU will be sent to the device (GPU) memory.

### An improved center star algorithm

As we discussed in “Center star strategy” section, a straightforward implementation of center star strategy is time-consuming mainly because its first stage. In spite of an improved method has been proposed by HAlign, which could substantially reduce the time of finding the center sequence, it still has a *O*(*mn* ^2^) time complexity. For large-scale datasets where *n*≫*m*, it would become the bottleneck. Thus in this paper, we propose a bitmap based algorithm to further optimize the center sequence selection process.

First, every sequence is partitioned into a series of disjoint segments. Each segment consists of 8 characters. We use 2 bits (binary number) to represent a character. So a segment needs 16 bits space, which then can be stored in one integer. An example is given in Table 2. Suppose characters ‘A’, ‘T’, ‘C’, ‘G’ are represented by binary numbers 00, 01, 10, and 11, respectively. The binary number of segment “ATCGCGAT” is 0001111011100001, which then is transformed into a decimal number 7905.

**Table 2:** Example of a segment. Convert the RNA/DNA segments to the decimal numbers

Segment	ATCGCGAT
Binary number	0001111011100001
Decimal number	7905

Second, an array of integers denoted as *Occ*[] is built for recording the time of occurrence of all segments. The decimal numbers of their represented segments are used as indexes. Therefore, the size of this array is 2^16^ (*Occ*[65535]) since the maximum decimal number of a segment is 2^16^. All elements of *Occ* is initialized with zero. Next, we look through all segments in each sequence and count the occurrences of them. For example, if a sequence has a segment “ATCGCGAT”, whose decimal number is 7905, then the value of *Occ*[7905] is increased by one. Notice that same segments in one sequence only count once.

Finally, we find the center sequence with *Occ*. We calculate a similarity score (*SS*) for each sequence by accumulate the occurrences of all its segments. Suppose a sequence contains *p* segments and the decimal numbers of these segments are *d*_1_,*d*_2_,…,*d*_*p*_, then its similarity score is: *SS*=*Occ*[*d*_1_]+*Occ*[*d*_2_]+⋯+*Occ*[*d*_*p*_]. After calculating all similarity scores, the sequence with a maximum *SS* is chosen to be the center sequence.

If there are *n* sequences with the average length of *m*, building the *Occ* array for one sequence needs a time of *O*(*m*). So the process incurs a time cost of *O*(*mn*) for *n* sequences. Besides, calculating a similarity score for one sequence needs to access *Occ* *m* times, so all *SSs* can be calculated in the time of *O*(*mn*). Therefore, the total time complexity of center sequence selection is *O*(*mn*), which is less than HAlign’s *O*(*mn* ^2^). In our experiments, the process of finding the center sequence can complete in a few seconds for a dataset with 500 thousands of sequences.

As discussed in “Center star strategy” section, we apply the dynamic programming method in the second phase of the center star strategy, which requires a time of *O*(*m* ^2^ *n*). In other words, the second step of CMSA, i.e. pairwise alignments, is now the most time-consuming phase. Therefore, we only focus on parallelizing the second step.

### Workload distribution

One of the key issues of a heterogeneous system is load balance. Since CPU and GPU differ greatly in computing capability, a heterogeneous system needs a way to estimate this differential to achieve the load balance. Suppose the execution time of CPU and GPU are *T*_1_ and *T*_2_, then the total time of the pairwise alignment is the maxmum value of *T*_1_ and *T*_2_. So the best performance is achieved when the computations of CPU and GPU are completely overlapped, which means *T*_1_=*T*_2_.

In CMSA, a pre-computation process is performed to decide how to distribute the workload for CPU and GPU. In this process, both CPU and GPU computes the same number of sequences (a small portion of input sequences). CMSA compares the execution time of CPU and GPU (denoted as *t*_1_ and *t*_2_) to calculate a ratio of computing capability *R*, $R = \frac {t_{1}}{t_{2}}$. According to this ratio, CMSA then assigns $\frac {n}{R+1}$ sequences to CPU and the rest $\frac {Rn}{R+1}$ sequences to GPU, where *n* is the number of input sequences.

### Parallel optimization of pairwise alignment

In the CPU end, OpenMP is used to accelerate the pairwise alignment in a coarse-grained manner. The computation of the DP matrix and the backtracking of score matrices are mapped onto different threads. In other words, each thread is responsible for aligning the center sequence with a different sequence. Threads are working independently, and each thread handles its own memory space including allocating and releasing the resources. The number of threads is usually set to the number of cores in the CPU.

Typical general-purpose graphics processors consist of multiple identical instances of computation units called Stream Multiprocessors (SM). SM is the unit of computation to which a group of threads, called thread blocks. A CUDA program consists of several *kernels*. When a kernel is launched, a two-level thread structure is generated. The top level is called *grid*, which consists of one or more *blocks*, denoted as *B*. Each block consists of the same number of threads, denoted as *T*. The whole block will be assigned to one SM.

Like the implementation on CPU, each thread in a kernel aligns one sequence with the center sequence, which means each kernel computes *B*×*T* sequences. As we discussed early, GPU handles $\frac {Rn}{R+1}$ sequences totally. So on the GPU end, $\frac {Rn}{(R+1)(BT)}$ kernels are executed. Since each kernel computes the same number of sequences and the DP matrices computed by each kernel are not used in the next kernel, we could recycle these memory resources. Before the first kernel is invoked, CMSA allocates the memory required for storing the DP matrices in one kernel. And when the last kernel finishes, the memory will be released. The DP matrix is stored in a one-dimensional way in the global memory of GPU. For example, there is a 12GB global memory. In theory, each kernel can simultaneously compute 53688 sequences of the length of 200 if each element in the DP matrix contains three short *short* type digital in this paper.

## Results and discussion

We evaluate CMSA using 16s rRNA sequences on a heterogeneous CPU/GPU workstation. In this section, we first introduce the experimental environments and then evaluate the efficiency and scalability of CMSA along with our bitmap based algorithm. Finally, we compare CMSA with some of state-of-the-art MSA tools.

### Experimental setup

#### Experimental platform

The experiments are carried out on a heterogeneous CPU/GPU platform, which has 32 GB RAM, an Intel Xeon E5-2620 2.4 GHz processor and an NVIDIA Tesla K40 graphic card. Centos 6.5 is installed and CUDA Toolkit 6.5 is used to compile the program. The CPU consists of 12 cores. The detailed specifications of Tesla K40 is shown in Table 3.

**Table 3:** GPU hardware specifications

Tesla K40	
CUDA Driver Version / Runtime Version	8.0 / 8.0
CUDA compute capability	3.5
CUDA cores	2880
GPU clock rate (MHz)	745
Total amount of global memory (GB)	12
Memory bandwidth (GB/s)	288
Shared memory size per block (bytes)	49152
Registers available per block	65536

#### Datasets

The BALiBASE is small and is suited only for protein alignment. As there is no benchmark datasets contain large-scale DNA/RNA sequences, we employ human mitochondrial genomes(mt genomes) and 16s rRNA. 16s rRNA sequences are often used to infer phylogenetic relationships and to distinguish species in microbial environmental genome analyses (Hao et al., 2011). All sequences are obtained from NCBI’s GenBank database (http://www.ncbi.nlm.nih.gov/pubmed). The mt genomes is a highly similar dataset. To address DNA/RNA sequences with low similarity, we also tested our program on 16s rRNA. We classified these 16s rRNA sequences into three datasets according to their average lengths, named as D1, D2 and D3, respectively, as shown in Table 4.

**Table 4:** Experimental datasets

Dataset	Average length	Num	File size
MT	16569	672	11 MB
D1	252	500000	183.8 MB
D2	490	500000	290.6 MB
D3	748	500000	414.3 MB
16s rRNA	1388	1011621	1.4 GB

#### Metrics

The sum-of-pairs (SP) score is often chosen to measure the alignment accuracy. The SP score is the sum of every pairwise alignment score from the MSA. But for large-scale datasets, it may be very large and exceeds the computer’s limitation. Thus we employ the average SP value, which is simply divided the SP value by the number of sequences, n. The average SP can also describe alignment performance. In the experimental tests, a program, “ *bali*_*score*”, downloaded from the Balibase benchmark (http://www.lbgi.fr/balibase/) was used to compare the alignment results.

#### Baselines

To show the efficiency and accuracy of CMSA, we compare CMSA with state-of-the-art MSA tools including Kalign, MAFFT and HAlign. Most of state-of-the-art MSA software cannot handle large-scale datasets. In order with data handling size, these tools are T-Coffee (small), CLUSTAL (medium), MAFFT (medium-large) and Kalign(large), as suggesting by EMBL-EBI. Therefore, the MAFFT, Kalign v2 is adopted. Besides, HAlign is the state-of-the-art software using center star strategy. Therefore, we use HAlign, MAFFT and Kalign v2 as benchmarks, and default parameters of Kalign v2, MAFFT and HAlign are used. For fairer comparison, all experiments are conducted on one node.

### Bitmap based algorithm for selecting the center sequence

As we discussed in “Center star strategy” section, both HAlign and CMSA are based on center star strategy. HAlign uses a tire-tree based algorithm to find the center sequence whereas CMSA uses a bitmap based algorithm. To evaluate our new proposed algorithm, we first compare the running time of the first stage of HAlign and CMSA. Then we perform the subsequent steps using the center sequence selected by HAlign and compare its results with ours. In addition to our own datasets, we also test HAlign and CMSA on the human mitochondrial genomes dataset(marked as MT), which is used in HAlign’s experiments. The human mitochondrial genome dataset is a highly similar dataset. It has a total of 672 human mitochondrial genomes shown in Table 4.

Table 5 shows the running time and SP score of HAlign and CMSA(CPU) based on different center sequence selection algorithms. For fairness, the HAlign was tested on only one node. The center sequence showed in the table is the zero-based index of sequences. As we can see, CMSA is much faster than HAlign in all experiments since our bitmap based algorithm has a lower time complexity (*O*(*mn*)). Also, HAlign runs out of memory when computing dataset D3 with 5000 sequences. When processing the dataset D2 with 1000 sequences and the dataset D3 with 1000 sequences, HAlign and CMSA find the same center sequence. Except these two tests, HAlign and CMSA reach a different result. And when inspecting the average SP score, CMSA performs better than HAlign. Besides, the better average SP score occurs with the datasets of high similarity. Thus we can conclude that our new algorithm used to find the center sequence is efficient and accurate with high and low similarity.

**Table 5:** The running time and SP score of single core HAlign and CMSA(CPU) based on different center sequence selection algorithms

Dataset	Num	Center sequence		Running time(s)								Average SP score	
		HAlign	CMSA	HAlign				CMSA				HAlign	CMSA
				Step1	Step2	Step3	Overall	Step1	Step2	Step3	Overall		
MT	672	16	479	88.19	33.40	21.11	142.70	0.80	43.40	0.50	44.70	0.977	0.987
	1000	912	575	2.22	0.42	0.24	2.88	0.05	0.40	0.03	0.48	0.549	0.570
D1	3000	912	575	23.17	2.75	0.77	26.69	0.13	1.20	0.10	1.43	0.550	0.588
	5000	3477	2266	67.95	2.10	1.28	71.33	0.16	2.15	0.23	2.54	0.492	0.523
	1000	158	158	6.93	0.52	0.99	8.44	0.07	1.40	0.10	1.57	0.508	0.548
D2	3000	181	1447	70.64	5.07	1.20	76.91	0.18	4.90	0.25	5.33	0.484	0.500
	5000	3533	4677	200.38	10.31	7.60	218.29	0.25	0.96	0.42	1.63	0.455	0.510
	1000	697	697	13.50	2.19	1.85	17.54s	0.12	4.22	0.17	4.15	0.513	0.540
D3	3000	2170	3217	125.06	2.19	1.85	129.10	0.24	13.44	0.34	14.02	0.527	0.528
	5000	2420	2992	351.49	8.40	9.75	369.64	0.37	22.13	0.60	23.10	0.518	0.523

### Efficiency and scalability

**Fig. 3:**
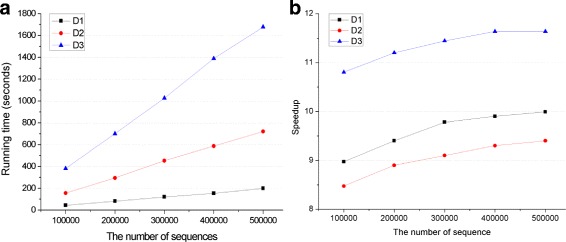
Experiments on datasets with different number of sequences. D1, D2, D3 represent three kinds of datasets described in Table 4. **a** Running time and **b** Speedup

As an indication of how CMSA scales with the size of dataset, Fig. 3 a shows the running time of CMSA on all three datasets described in Table 4. It’s clear that the longer the average length is, the more time it would cost. Moreover, in all three datasets, the running time goes up linearly as the number of sequences increases, which demonstrates a great scalability of CMSA. Figure 3 b shows the speedup of the same experiments. The best speedup is not achieved at first since with a low number of sequences, the runtime of the pre-compute and initialization makes up a considerable proportion. With the increase of the number of sequences, the real computation would dominate most of the running time, which in turn reports a better speedup.

We have test the CMSA (CPU/GPU) with different numbers of sequences(average length:252). Table 6 shows the workload ratio (R) described in “Workload distribution” section. From the table, the values of workload ratio are similar, and the average workload ratio of GPU and CPU is 1.420. We can confirm that CMSA has the good method of workload distribution for CPU and GPU.

**Table 6:** Workload radio for GPU and CPU

Dataset	Number	Workload radio
D1	100000	1.382
	200000	1.432
	300000	1.435
	400000	1.426
	500000	1.423

### Comparison with State-of-the-art tools

To show the efficiency and accuracy of CMSA, we compare CMSA with state-of-the-art MSA tools. In this setion, CMSA(CPU) and CMSA(CPU/GPU) are both tested.

Table 7 shows the time consumed for three datasets with different number of sequences computed. In our experiments, Kalign cannot handle datasets that consist of more than 100,000 sequences. MAFFT runs without a problem, but it takes too much time, e.g. 18 h for D1 with 100,000 sequences and more than 24 h for D2 and D3 with 100,000 sequences. So we don’t record the exact running time of CMSA for D2 and D3 with more than 100,000 sequences. In comparison, both HAlign and CMSA can handle all datasets in an acceptable time. Moreover, in all experiments, CMSA is the fastest one and also the one having the best scalability as the number of sequences increases. When computing D3, CMSA is 13× faster than HAlign when the dataset size is 10,000 and 24× faster when the size increases to 500,000.

**Table 7:** Running time of different MSA tools with different number of sequences and average length

Dataset	Number	Kalign	MAFFT	HAlign(one node)	CMSA(CPU)	CMSA(CPU/GPU)
D1	10000	20m39s	2m26s	39.99s	6.66s	7.71s
	100000	-	18h30m	5m59s	41.61s	44.32s
	500000	-	-	33m17s	3m15s	3m20s
D2	10000	52m28s	4m40s	1m36s	16.21s	17.19s
	100000	-	-	22m11s	2m14s	2m36s
	500000	-	-	2h15m	11m6s	12m1s
D3	10000	79m23s	8m59s	10m9s	45.01s	44.11s
	100000	-	-	15m27s	6m16s	6m21s
	500000	-	-	11h2m	30m28s	27m58s

Table 8 shows the comparison result of average SP scores for 16 s rRNA datasets. From Table 8, we can observe that MAFFT produced better alignment results than other state-of-the-art MSA softwares when addressing the large-scale datasets. The average SP of CMSA was lower than that of MAFFT and higher than that of HAlign. Therefore, we confirm the robustness of CMSA, whether with large-scale or small datasets.

**Table 8:** Average SP scores of different MSA tools with different number of sequences and average length

Dataset	Number	Kalign	MAFFT	HAlign(one node)	CMSA(CPU)	CMSA(CPU/GPU)
D1	10000	0.570	0.560	0.340	0.467	0.428
	100000	-	0.561	0.340	0.478	0.431
	500000	-	-	0.372	0.473	0.423
D2	10000	0.458	0.472	0.329	0.467	0.447
	100000	-	-	0.380	0.474	0.454
	500000	-	-	0.327	0.480	0.449
D3	10000	0.480	0.479	0.401	0.474	0.414
	100000	-	-	0.376	0.477	0.437
	500000	-	-	-	0.472	0.441

## Related work

There are a number of work on MSA problems and many parallel techniques as well as optimization methods have been proposed to accelerate MSA algorithms. In this section, we review them from two aspects.

**MSA software and algorithms.** MSA software can be classified into two categories based on their underlying algorithms: heuristic based or combinatorial optimization based. Many popular MSA tools like T-Coffee [17], CLUSTAL [7], Kalign [5] and MAFFT [6] are based on heuristic methods. T-Coffee can make accurate alignments of very divergent proteins but only for small sets of sequences, given its high computational cost. CLUSTAL is suitable for medium-large alignments. On a single machine, it is possible to take over an hour to compute 10,000 sequences with a more accurate method of CLUSTAL. Kalign is as accurate as other methods on small alignments, but is significantly more accurate when aligning large and distantly related sets of sequences. MAFFT uses fast fourier transforms, which can handle medium-large file sizes and align many thousands of sequences. ClustalW [18] has more than 52,400 citations now and is considered the most popular MSA tool. A commercial parallel version of ClustalW is designed for expensive SGI shared memory multiprocessor machines [19]. ClustalW-MPI [20] targets distributed memory workstation clusters using MPI but parallelize only Stages 1 and 3 of ClustalW. It achieves speedup of 4.3 using 16 processors on the 500-sequences test data. MSA-CUDA [21] parallelizes all three stages of the ClustalW processing pipeline using CUDA and achieves average speedup of 18.74 for average-length protein sequences compared to the sequential ClustalW. CUDA MAFFT [22] also uses CUDA to accelerate MAFFT that can achieve speedup up to 19.58 on a NVIDIA Tesla C2050 GPU compared to the sequential and multi-thread MAFFT.

**Center star algorithm.** The center star algorithm is a combinatorial optimization method and it is much more suited for aligning similar sequences. Then, K-band [2] is proposed to reduce the space and time cost of the pairwise alignment stage of the center star strategy. Based on the fact that for similar sequences, the backtracking often runs along the diagonal, so the lower left quarter and the upper right quarter in dynamic programming table are not taken into consideration. Therefore the K-band method only computes the band of which the width is k nearby the diagonal of the dynamic programming table. HAlign [9]then further optimized the center star algorithm with a trie-tree data structure, as we discussed in “Center star strategy” section. But this method still requires a time cost of *O*(*mn* ^2^) to find the center sequence, which is not efficient enough to handle large-scale datasets. Because of this, their Hadoop version skips the center sequence selection process and just designate the first sequence as the center sequence. Moreover, to our best knowledge, there are no work exist on accelerating the center star algorithm with CUDA enabled GPUs.

## Conclusion

In this paper, we designed CMSA, a robust and efficient MSA system for large-scale datasets on the heterogeneous CPU/GPU system. CMSA is based on an improved center star strategy, for which we proposed a novel bitmap based algorithm to find the center sequence. The new algorithm reduces the time complexity from *O*(*mn* ^2^) to *O*(*mn*). Moreover, CMSA is capable of aligning a large number of sequences with different lengths, which extends the generality of previous studies in MSA. In addition, to fully utilize the computing devices, CMSA takes co-run computation model so that the workloads are assigned and computed on both CPU and GPU devices simultaneously. Specifically, we proposed a pre-computation mechanism in CMSA to distribute workloads to CPU and GPU based on their computing capacity. Moreover, the more accurate mechanism will be future work to be performed for CMSA.

The experiment results demonstrated the efficiency and scalability of CMSA for large-scale datasets. CMSA achieved a speedup of 11 at best and can handle a large dataset with 500,000 sequences in half an hour. We also evaluated our center sequence selection algorithm. It is much faster and accurate that trie-tree based algorithm proposed in HAlign. Besides, we compared CMSA with some state-of-the-art MSA tools including Kalign, HAlign and MAFFT. In all our experiments, CMSA outperformed those software both in average SP scores and in the execution times.

## Availability and requirements

- **Project name**: CMSA
- **Project home page**: https://github.com/wangvsa/CMSA
- **Operating system(s)**: Linux 64-bit
- **Programming language**: C++, CUDA, OpenMP
- **Other requirements**: CUDA-capable GPU
- **license**: GUN GPL
- **Any restrictions to use by non-academics**: None
- The datasets used in this paper is available from: http://datamining.xmu.edu.cn/software/halign/and http://www.ncbi.nlm.nih.gov/pubmed.
- The program,“*bali*_*score*", is available from the Balibase bench-mark (http://www.lbgi.fr/balibase/).

## Abbreviations

CPU: Central processing unit
CUDA: Compute unified device architecture
GPU: Graphics processing unit
MPI: Message passing interface
MSA: Multiple sequence alignment
NCBI: National center for biotechnology information
OpenMP: Open multiprocessing
PCIe: Peripheral component interconnect express
pthread: POSIX thread
